# Isolated Unilateral Hydrosalpinx Torsion in a Post-Tubal Ligation Patient: A Case Report and Review of Literature

**DOI:** 10.7759/cureus.56351

**Published:** 2024-03-17

**Authors:** Shubhada Jajoo, Suhas Jajoo, Rucha Naval

**Affiliations:** 1 Obstetrics and Gynecology, Jawaharlal Nehru Medical College, Datta Meghe Institute of Higher Education and Research, Wardha, IND; 2 General Surgery, Jawaharlal Nehru Medical College, Datta Meghe Institute of Higher Education and Research, Wardha, IND; 3 Laparoscopic Surgery, Naval Multispeciality Hospital, Jalgaon, IND

**Keywords:** salpingectomy, ischaemic necrosis, fallopian tube torsion, hydrosalpinx, undiagnosed abdominal pain

## Abstract

Isolated tubal torsion of the hydrosalpinx is a rare occurrence with a varied clinical presentation, presenting a diagnostic challenge. We present a case involving the isolated torsion of the right hydrosalpinx in a 33-year patient with a history of bilateral tubal ligation who presented with an acute abdomen. Based on ultrasound and clinical findings, an initial diagnosis of ovarian torsion was considered. However, escalating pain severity led to diagnostic laparotomy, revealing torsion in the right hydrosalpinx. Subsequent right salpingectomy was done, and as the patient had undergone tubal ligation, preventive left salpingectomy was also performed. Both ovaries were preserved. The patient experienced an uneventful recovery. A literature review uncovered fewer than 50 reported cases of unilateral or bilateral isolated fallopian tube torsion post-tubal ligation. This case underscores the diagnostic challenges associated with isolated tubal torsion and emphasizes the crucial role of early surgical intervention in preventing morbidity and preserving ovaries.

## Introduction

Isolated torsion of the fallopian tube, without simultaneous torsion of the ovary, is an infrequent cause of gynecological emergencies and a severe cause of pelvic pain in women. It represents an uncommon source of abdominal pain in women, accounting for 1-3% of cases initially presumed to be ovarian torsion [1]. Bland-Sultan first described isolated tubal torsion without ovary torsion in 1890 [2]. Its estimated prevalence is one in 1.5 million women [3,4]. Many times, women are of reproductive age [1]. Swift diagnosis and early detorsion are paramount to salvage the fallopian tube whenever fertility preservation is required and to prevent advent complications [5]. Clinical presentation is nonspecific. Diagnostic symptoms, clinical signs, laboratory markers, and pathognomonic radiological features are usually not found in these cases [6].

The mechanism of tubal torsion involves the rotation of the tube around its long axis, leading to mechanical obstruction of adnexal veins and lymphatics, resulting in pelvic congestion, edema, and enlargement of fimbria. This all leads to necrosis of the fallopian tube [7]. Its etiology is mainly hydrosalpinx, haematosalpinx, and tubal ligation leading to hydrosalpinx. This is characterized by dilatation of the fallopian tube exceeding 2 cm, which is recognized as a risk factor for adnexal torsion [8]. Although tubal torsion is rare, early diagnosis is still important. Late diagnosis leads to necrosis and irreversible damage. Fertility-preserving surgeries are recommended unless the tube is wholly necrosed [9]. Laparoscopy is the gold standard for establishing diagnosis and treatment [4]. Here, we report a case of isolated torsion of a right hydrosalpinx in a 33-year-old patient with prior tubal ligation, preoperatively diagnosed as ovarian torsion.

## Case presentation

A 33-year-old female presented with lower abdominal pain on the right side, persisting for eight days and vomiting for four days. Her obstetric history included two prior lower segment cesarean sections, 11 and seven years ago, followed by tubal ligation seven years ago. She reported regular menstrual cycles, with the last menstrual period on December 10, 2023. Upon examination, vital signs were typical, but the abdomen was guarded, and tenderness was noted in the right iliac fossa. Pelvic examination revealed tenderness in the right fornix, with bimanual palpation identifying a cystic lesion measuring 5 cm x 5 cm in the right adnexa, separate from the uterus.

Laboratory results were within normal ranges, including hemoglobin, white cell counts, platelet counts, blood urea, serum creatinine, and liver function tests (Table 1).

**Table 1:** Laboratory investigation. Hb, hemoglobin; WBC, white blood count; SGOT, serum glutamic-oxaloacetic transaminase; SGPT, serum glutamate pyruvate transaminase; Na, sodium; K, potassium; CA-125, cancer antigen 125

Name of the investigation	Result	Reference value
Hb	11.3 g/dL	12 to 16 g/dL
Platelet count	344,000	>150,000
WBC	9,000	4,000 to 11,000
CA-125	6.5 U/mL	<30.2 U/mL
SGOT	34 u/lt	Upto 40 u/lt
SGPT	260 u/lt	Upto 40 u/lt
Na	138 mmol/L	135 to 148 mmol/L
K	4.5 mmol/L	3.8 to 5.2 mmol/L
Bilirubin	1 mg	Upto 1 mg
Blood sugar (R)	100 mg/dL	<140 mg/dL
Creatinine	1.04 mg/dL	0.6 to 1.1 mg/dL

**Figure 1:**
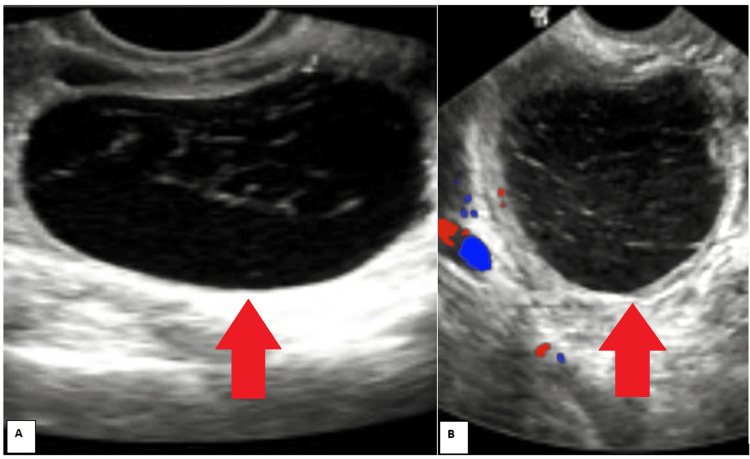
Preoperative ultrasound: (A) cystic lesion in the pelvis; (B) cystic lesion showing absence of vascularity (torsion).

Serum beta-human chorionic gonadotropin (beta-HCG) and urine pregnancy tests were negative, and the serum cancer antigen 125 (CA-125) level was average. Abdominal and pelvic ultrasound indicated a large extrinsic cyst from the right ovary, measuring 5 cm x 5 cm, with possible torsion. Douglas's pouch showed scanty fluid, while other organs appeared normal (Figure 1).

**Figure 2:**
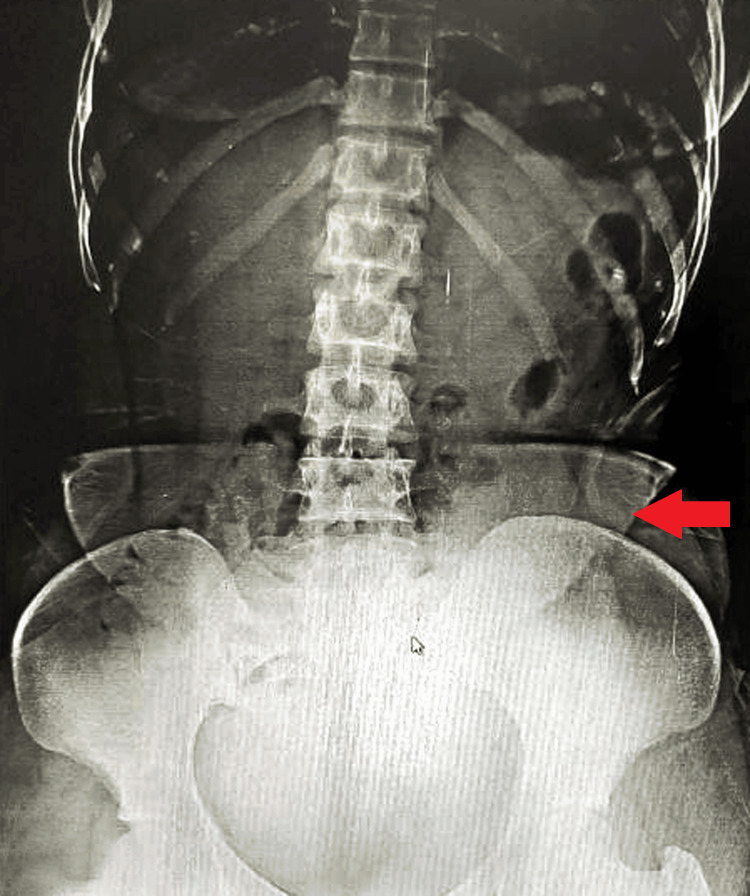
Abdomen in a standing position revealing no abnormalities.

An X-ray of the abdomen in a standing position revealed no abnormalities (Figure 2).

**Figure 3:**
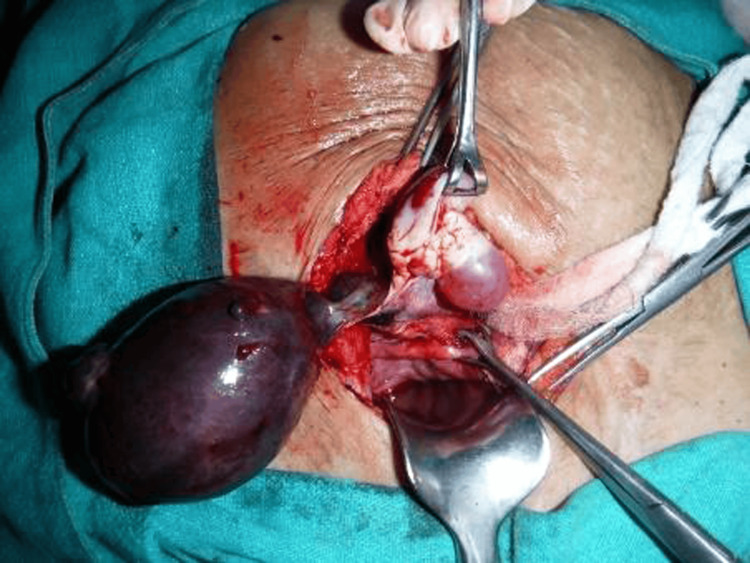
Isolated hydrosalpinx with normal ovary (normal ovary seen picked up with Babcock's forceps).

Laparotomy revealed a normal-sized uterus, a right-sided round ligament, and a normal ovary (Figure 3).

**Figure 4:**
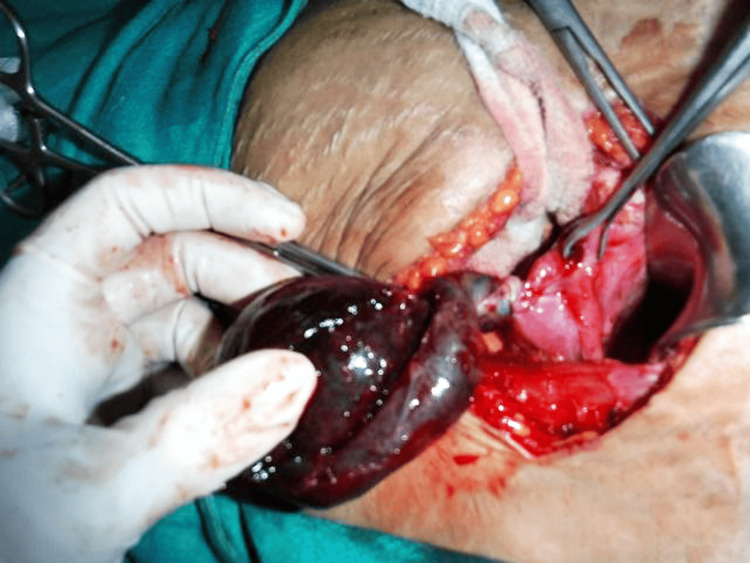
Isolated, twisted, black-colored hydrosalpinx seen picked up with a finger.

The right fallopian tube was enlarged, retort-shaped, and twisted at the corneal end, appearing as a black mass on the posterior right side of the uterus (Figure 4).

**Figure 5:**
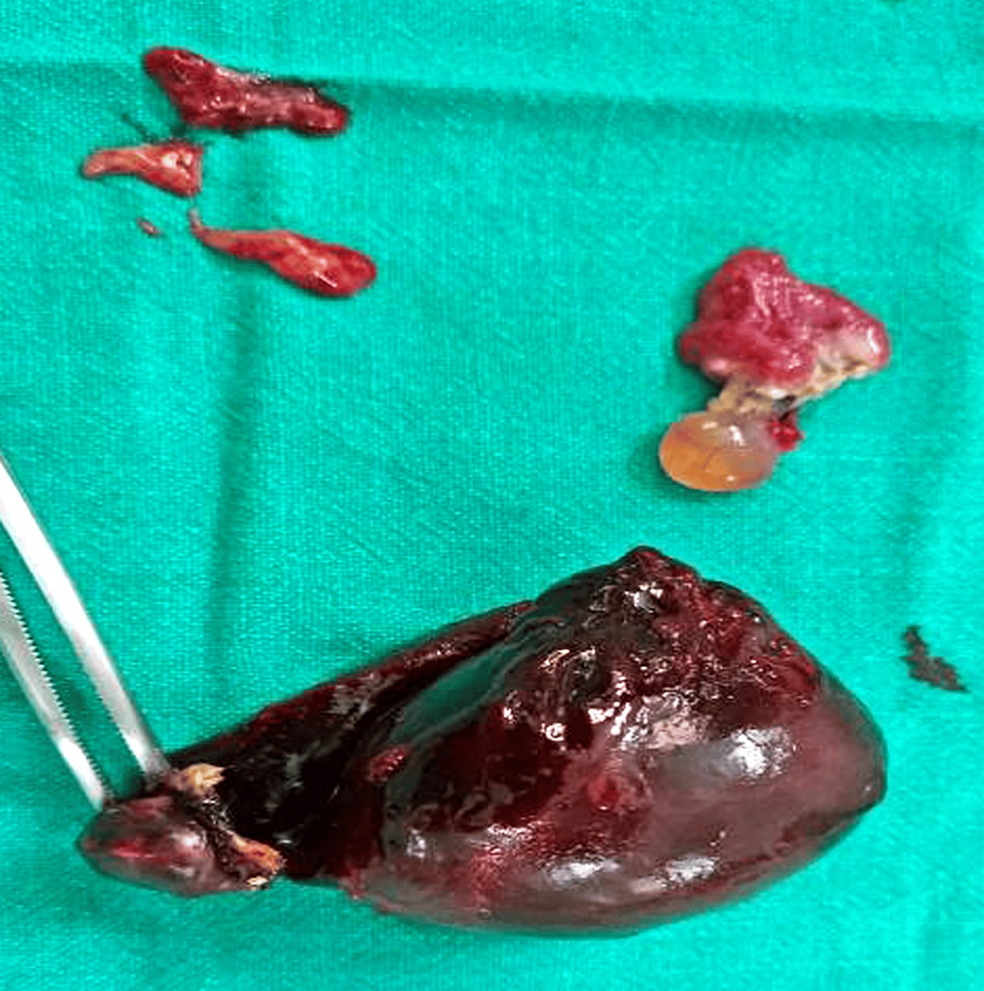
Retort-shaped necrotic hydrosalpinx seen after removal.

The mass revealed a normal ovary behind it. A right-sided salpingectomy was performed, revealing that the tube had been ligated in the past at the isthmus area, with the distal end dilated and containing paraphuron. A left-sided salpingectomy was also conducted. Both ovaries were preserved, and the postoperative course was uneventful. The specimen appeared necrotic, black-colored, and retort-shaped, measuring 7 cm x 6.5 cm x 3 cm (Figure 5).

**Figure 6:**
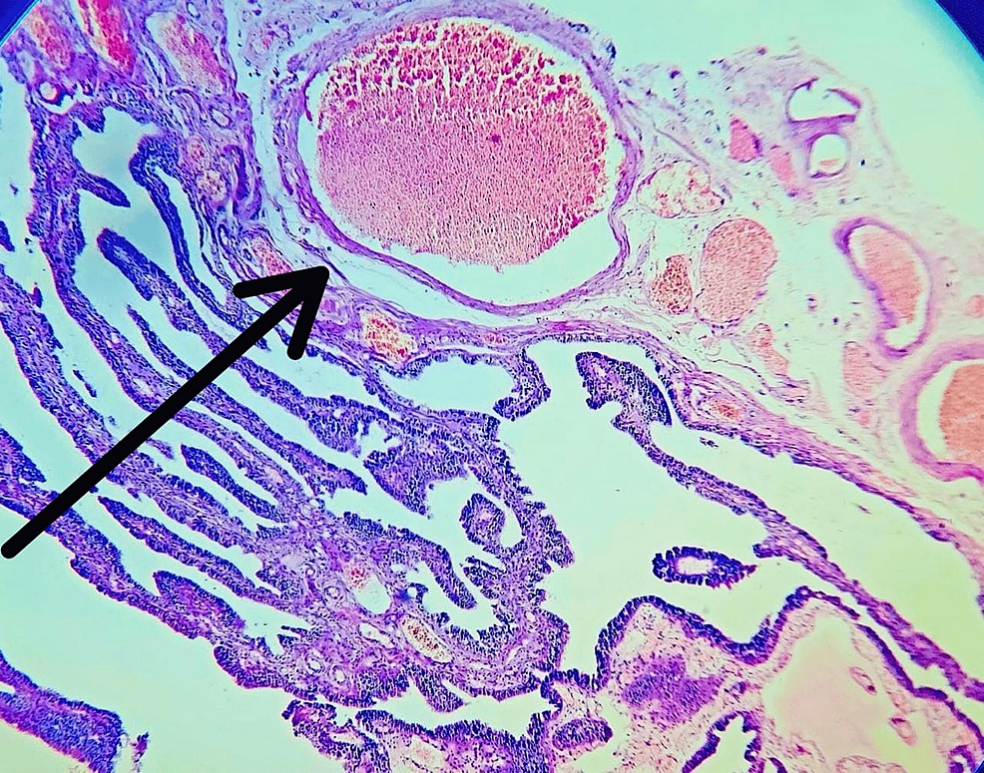
Right hematosalpinx measuring 7 cm x 6.5 cm x 3 cm.

The histopathological examination revealed a right hematosalpinx measuring 7 cm x 6.5 cm x 3 cm on gross examination (Figure 6). A microscopic examination showed salpingitis with necrosis.

## Discussion

Isolated tubal torsion is a rare event affecting women in all age groups [3]. In risk factors, Youssef et al. noted possible risk factors for fallopian tube torsion as internal and external risk factors [8]. Intrinsic factors are directly related to the fallopian tube, such as congenital anomalies, hydrosalpinx, tubal ligation, tubal neoplasm, etc. Extrinsic factors include ovarian or para-tubal masses, pregnancy, fibroids, and pelvic adhesions [10].

In our patient, the most probable factor is hydrosalpinx post-tubal ligation. It was present in the right fallopian tube during histopathological examination of the specimen inflammation with salpingitis. Our patient underwent tubal ligation, which is a rare risk factor for torsion. In the literature search, we found less than 50 cases of post-tubal ligation torsion since 1969, primarily because of the formation of hydrosalpinx. Other tubes did show hydrosalpinx, but still, salpingectomy was performed on the left side also. Both ovaries being normal, they were preserved [9].

If torsion is arrested before the onset of gangrenous changes, the tubes can be preserved in torsion cases. A study by Mazouni et al. emphasized that more than 10 hours between the onset of pain and surgery increases the risk of tubal necrosis [9,11]. The diagnosis of isolated tubal torsion by ultrasound is quite challenging. There should be high suspicion of this pathology when there is a normal-appearing ovary with ultrasound features of torsion [7]. However, clinical suspicion of this condition is often low, and detorsion may not be consistently successful, leading to delayed presentation and progression to hemorrhagic necrosis [12]. Salpingectomy is the preferred option in case of necrosis.

## Conclusions

Isolated tubal torsion is a rare but important differential diagnosis for women presenting with acute abdomen with a cystic mass. In our case, a diagnosis of adnexal mass was made, and isolated right fallopian tube torsion was found at laparotomy. As this was a case of tubal ligation with the removal of twisted hydrosalpinx on the right side, an a-sided salpingectomy was performed. Otherwise, conservative management must be the plan for women of childbearing age where fertility preservation is required. Clinical presentation is often nonspecific, and imaging has low diagnostic accuracy. Increased awareness of this entity can help prompt surgical intervention and preservation of the fallopian tube. Only long-term follow-up with a vast population can define a standardized treatment.
